# G Protein-Coupled Receptor 43 Modulates Neutrophil Recruitment during Acute Inflammation

**DOI:** 10.1371/journal.pone.0163750

**Published:** 2016-09-22

**Authors:** Marjon E. Kamp, Raymond Shim, Alyce J. Nicholls, Ana Carolina Oliveira, Linda J. Mason, Lauren Binge, Charles R. Mackay, Connie H. Y. Wong

**Affiliations:** 1 School of Biomedical Sciences, Monash University, Victoria, Australia; 2 Centre for Inflammatory Diseases, Department of Medicine, School of Clinical Sciences, Monash University, Victoria, Australia; 3 Laboratório de Imunologia Molecular, Instituto de Biofisica Carlos Chagas Filho, Universidade Federal do Rio de Janeiro, Rio de Janeiro, Brazil; Ludwig-Maximilians-Universitat Munchen, GERMANY

## Abstract

Fermentation of dietary fibre in the gut yields large amounts of short chain fatty acids (SCFAs). SCFAs can impart biological responses in cells through their engagement of ‘metabolite-sensing’ G protein-coupled receptors (GPCRs). One of the main SCFA receptors, GPR43, is highly expressed by neutrophils, which suggests that the actions of GPR43 and dietary fibre intake may affect neutrophil recruitment during inflammatory responses *in vivo*. Using intravital imaging of the small intestine, we found greater intravascular neutrophil rolling and adhesion in *Gpr43*^*−/−*^mice in response to LPS at 1 h. After 4 h of LPS challenge, the intravascular rolling velocity of GPR43-deficient neutrophils was reduced significantly and increased numbers of neutrophils were found in the lamina propria of *Gpr43*^*−/−*^mice. Additionally, GPR43-deficient leukocytes demonstrated exacerbated migration into the peritoneal cavity following fMLP challenge. The fMLP-induced neutrophil migration was significantly suppressed in wildtype mice that were treated with acetate, but not in *Gpr43*^*−/−*^mice, strongly suggesting a role for SCFAs in modulating neutrophil migration via GPR43. Indeed, neutrophils of no fibre-fed wildtype mice exhibited elevated migratory behaviour compared to normal chow-fed wildtype mice. Interestingly, this elevated migration could also be reproduced through simple transfer of a no fibre microbiota into germ-free mice, suggesting that the composition and function of microbiota stemming from a no fibre diet mediated the changes in neutrophil migration. Therefore, GPR43 and a microbiota composition that allows for SCFA production function to modulate neutrophil recruitment during inflammatory responses.

## Introduction

Microbes in the gut were once considered harmful or pathogenic, however it is now recognized that commensal bacteria and their metabolites are critical in regulating the development, homeostasis and function of innate and adaptive immunity [1–4]. In addition to providing protection against pathobionts in the gastrointestinal tract, and contributing in vitamin synthesis, the gut microbiota is essential for the fermentation of dietary fibre to yield short chain fatty acids (SCFAs) [5]. SCFAs, including acetate, proprionate and butyrate, are emerging as important regulators of host inflammatory responses [6]. Accumulating evidence suggests that a diet lacking sufficient amounts of fibre can result in an altered gut microbiota composition and a distorted state of intestinal wellbeing [7], contributing to local and systemic inflammatory diseases [8]. In fact, the dramatic increase in the incidence of inflammatory bowel disease (IBD), autoimmunity and allergy in Westernized countries parallels a decrease in the consumption of fibre and indigestible starches [6]. However, the precise contribution of diet, particularly fibre and SCFAs, in the modulation of host inflammatory responses remains poorly defined, and possible molecular mechanisms are now emerging [9–11].

Some of the protective effects of SCFAs produced by gut microbial fermentation of dietary fibre have been suggested to occur through the direct actions on G protein-coupled receptors (GPCRs) expressed on leukocytes [12,13]. SCFAs produced by gut microbial fermentation of dietary fibre and its associated receptors have recently been proposed as the potential link between diet, gut microbiota and the body's inflammatory response [14]. In particular, GPR43 is highly expressed on innate immune cells, especially neutrophils, and functions as an “anti-inflammatory chemoattractant receptor” for SCFAs [15]. The GPR43-dependent effects of SCFAs are necessary for the normal resolution of intestinal immune responses, with *Gpr43*^*−/−*^mice showing exacerbated inflammation in dextran sulfate sodium- (DSS-) induced colitis [15]. One of the major obstacles to unraveling the function of SCFAs and GPR43 *in vivo* is the sheer complexity of the cells and mechanisms that contribute to gut homeostasis and immune regulation. Despite recent research revealing a role for dietary fibre or SCFA in immune modulation of chronic diseases such as colitis, arthritis and asthma [9,14–16], the impact that fibre and its metabolites have on the initial events of neutrophil recruitment during intestinal inflammation *in vivo* remains unclear.

Inflammation begins with the recruitment of early responding leukocytes, such as neutrophils, along a chemotactic gradient to the site of injury or damage [17]. The leukocyte recruitment cascade entails the initial capture of intravascular leukocytes onto the endothelium via selectin binding, slow rolling, firm adhesion assisted by integrins, and finally the transmigration of the leukocytes out of the vasculature into the relevant tissues [18]. In human IBD patients, pharmacological approaches that target specific steps of the leukocyte recruitment cascade are emerging as effective therapeutic strategies [19,20]. Being highly motile, neutrophils are essential for the initial recognition of infection and inflammation, but they are also important for the further recruitment of other leukocytes to establish an appropriate and coordinated immune response. Indeed, neutrophils are armed with antimicrobial agents to combat translocated pathogens that gain entry to the intestinal epithelium [21]. Therefore, it is becoming clear that simply removing neutrophils or halting their activation following inflammation is not the answer for therapeutic targeting. Instead, proper control of neutrophil trafficking and function are important for efficient and appropriate immune responses during intestinal inflammation. In this study, we investigated the effects of GPR43 and dietary fibre on neutrophil recruitment following various models of acute inflammation. Using intravital microscopy of the small intestine and *in vitro* transwell chemotactic assays, we demonstrated that the absence of GPR43 or a fibre-deficient diet results in exacerbated neutrophil recruitment and migration in response to inflammation.

## Methods and Materials

### Mice

C57BL/6J and *Gpr43*^*−/−*^age and sex-matched male mice of 6–8 weeks old (backcrossed to C57BL/6J > 13 generations) were obtained from Monash Animal Services and housed under specified pathogen-free (SPF) conditions in Monash University Animal Research Laboratories. Germ-free (GF) male C57BL/6J mice at 7 weeks old were obtained from Walter and Eliza Hall Institute of Medical Research Animal Facility. Following transportation, mice were acclimatized for a minimum period of 7 days before use. Mice were housed in a 12-hour light-dark cycle in a temperature controlled environment with free access to food and water. Where indicated, mice were supplemented with 200mM acetate in the drinking water, or removed from their normal chow and fed either control diet (Ctrl; AIN93G, consisting of 4.7% crude fibre; Specialty Feeds) or no fibre diet (NF; SF09-028, consisting 0% fibre, modified from AIN93G) for 2 weeks prior to experimentation. All animal experiments were approved by the Monash University Animal Ethics Committee.

### Treatments

To induce an acute inflammatory response, mice were injected intraperitoneally with either 0.5 mg/kg lipopolysaccharide (LPS) from *E*.*coli* (Sigma) or 100 μg/kg N-Formylmethionyl-leucyl-phenylalanine (fMLP, Sigma). Sterile saline was used as the control.

### Neutrophil isolation

At specific time points, mice were deeply anaesthetized with isoflurane and subsequently killed via cervical dislocation. Bone marrow leukocytes were isolated from the femur and tibias, filtered with 70 μm filter and layered onto Ficoll (GE Healthcare), spun for 20 mins at 1000*g* at room temperature without brake. Neutrophils were enriched and collected from the interface.

### Neutrophil respiratory burst assay

Neutrophils were then divided into 100ul samples and incubated with dihydrorhodamine (DHR) 123 in a 37°C water bath for 15 minutes. Phorbol myristate acetate (PMA) assay reagent was then added to cells and incubated for up to 30 minutes in a 37°C water bath. PBS was used in the place of PMA for control samples. Following incubation, L-selectin expression on neutrophils were labelled with APC-conjugated anti-mouse L-selectin (clone MEL-14, BD Bioscience) and BV510-conjugated anti-mouse Ly6G (clone 1A8, Biolegend). A Navios flow cytometer was then used to examine and measure the fluorescence of cells. FlowJo was then used to gate the neutrophil cell population and determine the median fluorescence intensity for DHR 123 and L-selectin on Ly6G^+^ neutrophils.

### Neutrophil chemotaxis assay

For the transwell chemotaxis assay, 4 x 10^5^ neutrophils were loaded into the top chamber of each well (Corning Life Sciences), with the bottom chambers containing various concentrations of mouse recombinant CXCL1 (Biolegend, 10^−11^–10^−7^ M), fMLP (Sigma, 10^−11^–10^−7^ M) and LPS (Sigma, 10^−9^–10^−5^ g/ml). The neutrophils were allowed to migrate for 3 h at 37°C in a 5% CO_2_ incubator. The number of neutrophils migrated to the bottom chamber of the transwell was obtained using an LSRII or Fortessa cytometer (BD Biosciences) at the Monash FlowCore Cytometry Facility, and analysed using FlowJo software.

### Intravital microscopy of the gut

At 1 or 4 h post-LPS, mice were anesthetized with a cocktail of ketamine: xylazine (200 mg/kg: 10 mg/kg) via intraperitoneal injection. The tail vein was cannulated to administer fluorescently labelled antibodies or to add additional anaesthetic, if required. The mouse was positioned on its back and the abdomen was opened by a midline incision. Part of the small intestine (jejunum) was externalised onto an imaging board and secured in place using kimwipes soaked in saline solution. Cells and tissue were stained by intravenous (IV) injection of 2.5μg of FITC-conjugated anti-mouse CD31 (clone 390, eBioscience) to label the vasculature, 1 μg PE-conjugated anti-mouse Ly6G (clone 1A8, BD Biosciences) to label neutrophils and 1 μg APC-conjugated anti-mouse F4/80 (clone BM8, eBioscience) to label macrophages. Post-capillary venules were imaged using Andor Spinning Disk Confocal microscopy at the Monash Micro Imaging Facility with an Olympus IX70 microscope. Movies were captured at 20x magnification with an NA of 0.7, using an argon/krypton laser. FITC, PE and APC were excited at 488/6 nm, 567/15 nm and 650/13 nm, respectively; and were detected using a 530/60 nm and 624/40 nm band pass filter and a 647 nm long pass filter, respectively. Exposure time for FITC, PE and APC was 300, 200 and 300ms, respectively. Movies acquired contained 100 frames for each fluorophore. Three or more fields of views were randomly selected and captured as movies for each mouse.

### Image analysis

Microscopy videos were analysed using ImageJ 1.48 software by converting to a hyperstack, and optimizing brightness and contrast levels. Movies in this study were made by merging the different layers, and subsequently saving it in.avi format. Rolling cells were counted as the amount of cells slowly moving through the vessel (more than 30 seconds to pass through the entire vessel), expressed relative to time and length of the vessel. Rolling velocity was calculated by the distance a cell had travelled over the time it took, expressed relative to speed. Adherent cells were counted as cells stationary at the same place for more than 30 seconds, expressed relative to time and surface area of the vessel.

### Histology

The small intestine was isolated, washed in PBS and cut into three segments, namely duodenum, jejunum and ileum, to fix for 24 h in 10% formaldehyde. Paraffin sections from fixed segments were cut into 10 μm sections and prepared to be stained with Haematoxylin and Eosin (H&E). Briefly, sections were immersed in acidified Haematoxylin for 1 min, and rinsed with tap water. The sections were stained using Eosin for 1–2 mins followed by washing. Ascending alcohol solutions (50%, 70%, 80%, 95% x 2, and 100% x 2) were applied to dehydrate tissue sections, and cleared using xylene. Sections were viewed using an Olympus Provis AX70 widefield microscope and histological quantification of neutrophil number per segment was performed using at least five transverse sections per mouse in a blinded procedure.

### Acute peritonitis model

To induce acute peritonitis in mice, we utilized the cecal ligation and puncture (CLP) technique. Briefly, mice were anesthetized with a cocktail of ketamine: xylazine (200 mg/kg: 10 mg/kg) via intraperitoneal injection. The mouse was positioned on its back and the abdomen was opened by a small midline incision. The cecum was identified and ligated 10 mm above the distal ending and punctured twice with a 26 G needle to achieve a sub-lethal CLP. The wound on the abdomen of the mouse was sutured and the mouse was then transferred onto a heat pad to maintain its body temperature at 37°C. The post-CLP mice received 1 ml of saline subcutaneously for volume substitution. At 24 h after CLP, mice were put into deep anaesthesia with isoflurane and subsequently killed via cervical dislocation. Peritoneal lavage fluid was collected and the cell numbers were counted.

### Microbiota transfer to Germ free mice

C57BL/6 SPF mice were fed with either a Ctrl or NF diet for 2 weeks. After this, 5 pellets of faeces were collected, diluted in 3 ml of PBS, vortexed and the suspension was filtered through 70 μm filters. This material was immediately transferred by oral gavage to GF mice (200 μl per mouse) kept on a normal chow diet. This procedure was repeated, one week later. At the end of the second week (after 2 transfers), GF mice were culled and bone marrow leukocytes from the femur and tibias were collected. Donor mice and conventionalised GF mice were analysed in parallel.

### Statistical analyses

All values were expressed as mean ± standard error of the mean (SEM). Data were compared either by unpaired two-tailed Student’s *t*-test, one-way or 2-way ANOVA with Bonferroni multiple comparisons post hoc test. P-values < 0.05 were considered statistically significant.

## Results

**Fig 1:**
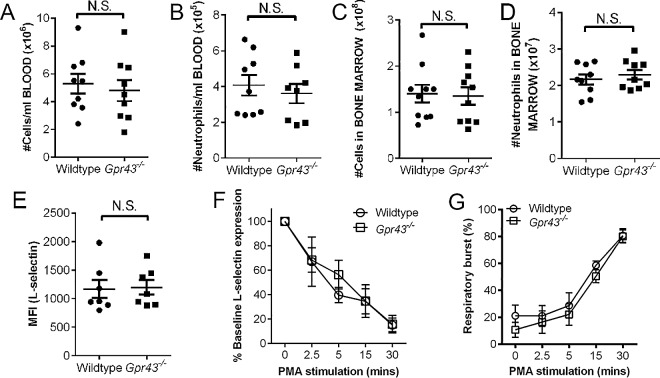
Baseline parameters are comparable between wildtype and *Gpr43*^*−/−*^mice. The number of circulating leukocytes (**A**) and neutrophils (**B**) in the peripheral blood of wildtype and *Gpr43*^*−/−*^mice were quantitated. Bone marrow leukocytes (**C**) and neutrophils (**D**) isolated from wildtype and *Gpr43*^*−/−*^mice were quantitated. N ≥ 8 per group. Baseline cell surface expression of L-selectin (**E**), and PMA-induced L-selectin shedding (**F**) and respiratory burst (**G**) was measured and compared between wildtype and *Gpr43*^*−/−*^bone marrow neutrophils. N ≥ 4 mice per group or independent *in vitro* experiments performed in duplicates, N.S. denote not statistical significant, *t* test.

Previous studies have shown the immunomodulating effects of SCFAs and GPR43 in chronic inflammatory responses, however the role of GPR43 in the initial events of neutrophil recruitment during acute inflammation remains unclear. Under basal conditions, we found no differences in the number of circulating leukocytes and neutrophils in the peripheral blood (**Fig 1A and 1B**) and in the bone marrow (**Fig 1C and 1D**) between wildtype and *Gpr43*^*−/−*^mice. In addition, the cell surface expression of L-selectin on isolated bone marrow neutrophils was also comparable between wildtype and *Gpr43*^*−/−*^mice (**Fig 1E**). Upon stimulation, the PMA-induced L-selectin shedding (**Fig 1F**) and respiratory burst (**Fig 1G**) in bone marrow neutrophils isolated from wildtype and *Gpr43*^*−/−*^mice were similar. Therefore, these data suggest there is no overt differences between wildtype and *Gpr43*^*−/−*^neutrophils at basal conditions.

**Fig 2:**
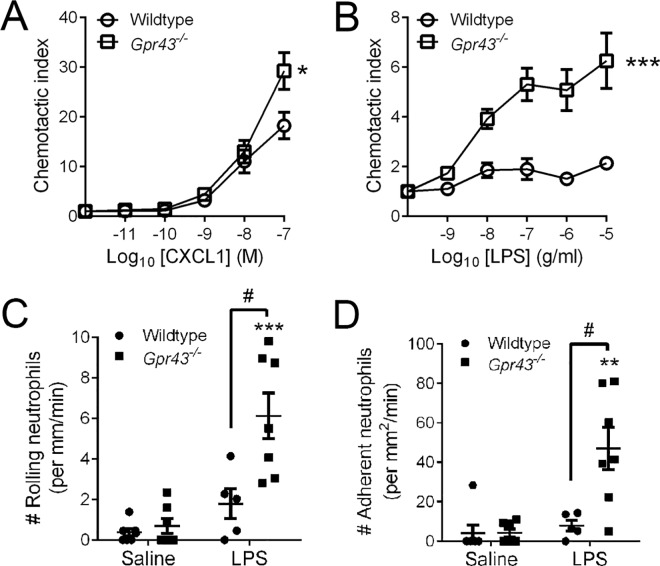
GPR43 deficiency induces migration, accelerated neutrophil rolling and adhesion following 1 h of LPS challenge. Neutrophils were isolated from the bone marrow of wildtype and *Gpr43*^*−/−*^mice, and analysed for chemotaxis towards increasing concentration of CXCL1 (**A**) and LPS (**B**). N ≥ 4 independent *in vitro* experiments performed in duplicates, ***p < 0.001, *p < 0.05 vs Wildtype, 2-way ANOVA. Intravital microscopy was utilised to visualise and quantitate the number of intravascular neutrophils that were rolling (**C**) or adherent (**D**) following 1 h of saline or LPS challenge in wildtype and *Gpr43*^*−/−*^mice. N ≥ 5 mice per group, ***p < 0.001, **p < 0.01 vs corresponding Saline group, *t* test. #*p* < 0.05 vs Wildtype, *t* test.

Next, the isolated bone marrow neutrophils from untreated wildtype and *Gpr43*^*−/−*^mice were examined for their migratory function in an *in vitro* chemotaxis assay. We found that neutrophils from *Gpr43*^*−/−*^mice showed increased chemotaxis towards the classic chemoattractant, CXCL1 (**Fig 2A**). The lack of migration by wildtype neutrophils towards a gradient of LPS suggests that LPS is not a typical chemoattractant, however we observed significantly elevated LPS responsiveness by GPR43-deficient neutrophils (**Fig 2B**). These results are consistent with published reports that demonstrated GPR43-deficient neutrophils showed elevated migratory behaviour towards bacterial products (fMLP) and to the complement fragment C5a [15].

The ideal approach for studying leukocyte trafficking and recruitment *in vivo* is via imaging with intravital microscopy. In this technique, blood vessels are visualised in live animals, and leukocytes are examined microscopically while they undergo the dynamic interactions with the vessel wall required to exit the bloodstream [22]. Using this technique, we investigated the effect of GPR43 on the intravascular recruitment of neutrophils following an endotoxin model of acute inflammation (LPS) *in vivo*. We found no difference in neutrophil trafficking between wildtype and *Gpr43*^*−/−*^mice under basal conditions, but GPR43-dependent alterations in neutrophil recruitment were observed as early as 1 h after LPS challenge. Specifically, there was a significant increase in LPS-induced neutrophil rolling (**Fig 2C**) and adhesion (**Fig 2D**) in *Gpr43*^*−/−*^mice compared to their wildtype counterparts at 1 h post-LPS. Taken together, these *in vitro* and *in vivo* data suggests that GPR43 is critical in modulating and restricting appropriate migration of neutrophils towards an inflammatory stimulus.

**Fig 3:**
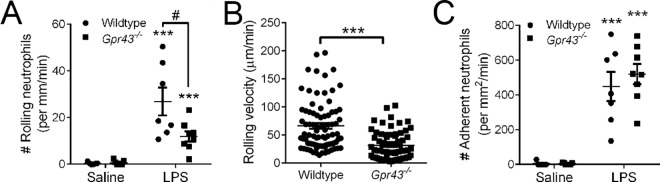
GPR43-deficient neutrophils display slow rolling following 4 h of LPS challenge. Intravital microscopy was utilised to visualise and quantitate the number of intravascular neutrophils that were rolling (**A**), and their rolling velocity (**B**) or adherent (**C**) following 4 h of saline or LPS challenge in wildtype and *Gpr43*^*−/−*^mice. N ≥ 5 mice per group, ***p < 0.001 vs corresponding Saline group, *t* test. # *p* < 0.05 vs Wildtype, *t* test.

**Fig 4:**
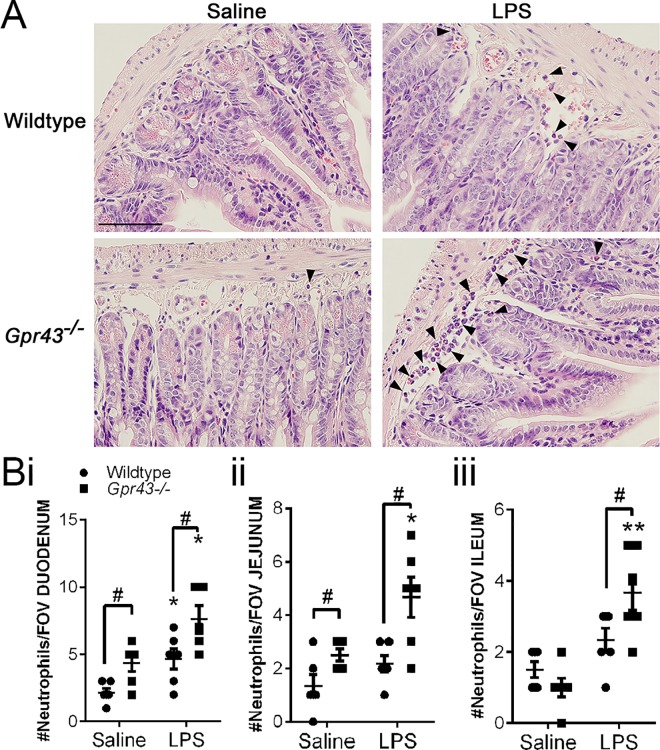
Exacerbated intestinal inflammation in *Gpr43*^*−/−*^mice in response to LPS. (**A**) Representative histological images of jejunum of wildtype and *Gpr43*^*−/−*^mice following 4 h of saline or LPS challenge. Neutrophils are denoted by the black arrowheads. Scale bar = 100 μm. (**B**) Histological quantification of neutrophils in the (**i**) duodenum, (**ii**) jejunum and (**iii**) ileum of wildtype and *Gpr43*^*−/−*^mice following 4 h of saline or LPS challenge. N ≥ 5 mice per group, **p < 0.01, *p < 0.05 vs corresponding Saline group, *t* test. #*p* < 0.05 vs Wildtype, *t* test.

We next examined the effect of GPR43 on the intravascular recruitment of neutrophils following 4 h of LPS challenge *in vivo*. Despite the significant increase in neutrophil recruitment at this later time point following LPS challenge in both wildtype and *Gpr43*^*−/−*^mice, the number of rolling neutrophils in *Gpr43*^*−/−*^mice at 4 h after LPS was significantly reduced compared to the LPS-treated wildtype mice (**Fig 3A**). There was a notable difference in the behaviour of intravascular neutrophils in wildtype and *Gpr43*^*−/−*^mice at this time point of acute inflammation (**S1 and S2 Movies**). The rolling velocity of neutrophils in post-LPS *Gpr43*^*−/−*^mice was significantly lower than their wildtype counterparts (**Fig 3B**), suggesting that the GPR43-deficient neutrophils have prolonged interactions with the endothelium following LPS. Despite this, the number of adhering neutrophils (completely stationary cells) was not different between wildtype and *Gpr43*^*−/−*^mice at 4 h post-LPS challenge (**Fig 3C**). The observation of increased migration towards LPS *in vitro*, and reduced rolling velocity but comparable amount of adhering neutrophils in *Gpr43*^*−/−*^mice at 4 h post-LPS *in vivo* led us to propose that GPR43-deficient neutrophils are highly responsive to LPS and actively transmigrating into the lamina propria of the small intestine. Therefore, we performed histological studies and quantified the number of neutrophils found in the lamina propria of the small intestine following 4 h of LPS challenge (**Fig 4A**). Under basal conditions, we found an elevated number of neutrophils in the duodenum (**Fig 4Bi**) and jejunum (**Fig 4Bii**) of saline-treated *Gpr43*^*−/−*^mice compared to their wildtype counterparts. Following 4 h LPS challenge, we observed an exacerbated elevation of neutrophils in the duodenum (**Fig 4Bi**), jejunum (**Fig 4Bii**) and ileum (**Fig 4Biii**) of *Gpr43*^*−/−*^mice.

**Fig 5:**
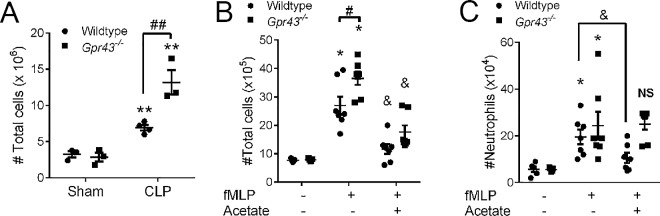
Acetate reduces neutrophil migration via a mechanism mediated by GPR43. The total number of leukocytes in the peritoneal cavity was quantified in wildtype and *Gpr43*^*−/−*^mice at 24 h following sham or cecal ligation and puncture (CLP; (**A**)), or at 2 h after saline, fMLP or fMLP + acetate (**B**). The peritoneal neutrophil numbers of acetate-treated wildtype and *Gpr43*^*−/−*^mice at 2 h after saline or fMLP treatment were also measured (**C**). N ≥ 3 mice per group, **p < 0.01, *p < 0.05 vs corresponding Sham or Saline group, *t* test. ##*p* < 0.01, #*p* < 0.05 vs treated Wildtype, *t* test. *&p <* 0.05 vs fMLP-treated Wildtype, *t* test. N.S denotes not statistically significant vs fMLP-treated *Gpr43*^*−/−*^.

We next examined the contribution of GPR43 in a mouse model of acute peritonitis. Under basal conditions, we found no difference in the number of leukocytes in the peritoneal cavity between saline-treated wildtype and *Gpr43*^*−/−*^mice (**Fig 5A**). In wildtype mice, CLP resulted in a significant increase in the number of leukocytes recruited into the peritoneal cavity, a response which was significantly exacerbated in *Gpr43*^*−/−*^mice (**Fig 5A**). Similarly, leukocyte recruitment into the peritoneal cavity dramatically increased for both strains of mice at 2 h after fMLP challenge, but fMLP-treated *Gpr43*^*−/−*^mice demonstrated a further elevated response (**Fig 5B**). In this model of inflammation, the level of leukocyte recruitment following fMLP was significantly reduced with the administration of acetate in the drinking water (**Fig 5B**). Acetate is a prominent SCFA that is generated from fermentation of dietary fibre by the host microbiota. Intriguingly, administration of acetate was sufficient to lower neutrophil recruitment into the peritoneal cavity in post-fMLP wildtype mice (**Fig 5C**). However, this acetate-induced anti-inflammatory effect after fMLP challenge was absent in *Gpr43*^*−/−*^mice, strongly suggesting that acetate serves to regulate or suppress excessive neutrophil recruitment and transmigration in a GPR43-mediated manner following an inflammatory stimulus.

**Fig 6:**
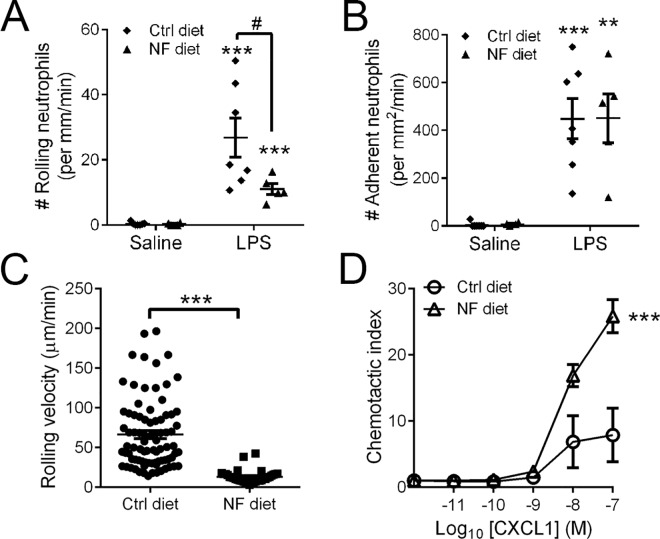
No fibre diet prolongs intravascular rolling of neutrophils post-LPS. Intravital microscopy was utilised to visualise and quantitate the number of intravascular neutrophils that were rolling (**A**) or adherent (**B**) following 4 h of saline or LPS challenge. (**C**) Neutrophil rolling velocity was calculated in Ctrl-fed and NF-fed mice at 4 h after LPS. N ≥ 5 per group, ***p < 0.001, **p < 0.01 vs corresponding Saline group, *t* test. #*p* < 0.05 vs Ctrl post-LPS, *t* test. Neutrophils were isolated from the bone marrow of mice fed on Ctrl or NF diet for 2 weeks, and analysed for chemotaxis towards increasing concentration of CXCL1 (**D**). N ≥ 4 independent *in vitro* experiments performed in duplicates, ***p < 0.001 vs Ctrl diet, 2-way ANOVA.

Prior to this study, it was unknown whether alterations in dietary fibre intake could mediate direct changes in neutrophil recruitment during an acute inflammatory response. Therefore, to investigate this, we fed wildtype mice that were born and raised in an SPF environment with Ctrl or NF diet for 2 weeks. Under basal conditions, we found no difference in neutrophil trafficking between wildtype mice fed with Ctrl or NF diet (**Fig 6A and 6B**). However, we observed markedly elevated neutrophil rolling (**Fig 6A**) and adhesion (**Fig 6B**) at 4 h after LPS challenge. Compared to the Ctrl-fed mice, there was significantly reduced number of rolling neutrophils in NF-fed mice at 4 h post-LPS (**Fig 6A**). Despite the lower level of neutrophil rolling in NF-fed mice at 4 h, these neutrophils were rolling at a markedly reduced velocity (**Fig 6C**). Moreover, we observed comparable neutrophil adhesion between Ctrl-fed and NF-fed mice after 4 h LPS (**Fig 6B**). Interestingly, these *in vivo* neutrophil recruitment data from post-LPS NF-fed wildtype mice are very similar to those observed in post-LPS *Gpr43*^*−/−*^mice (**Fig 3**). To further investigate the effect of dietary fibre intake on neutrophil function, we examined chemotactic function of bone marrow neutrophils isolated from mice that were either fed a Ctrl or NF diet. Neutrophils isolated from NF-fed mice showed significantly increased migratory function towards a gradient of the chemokine CXCL1 (**Fig 6D**). Together, these results suggest that a gut microbiota shaped by NF feeding, a microbiota that lack the ability to produce SCFAs, promotes 1) increased neutrophil chemotaxis and, 2) slowing down of neutrophils as they roll along the intestinal microvasculature during an inflammatory response.

**Fig 7:**
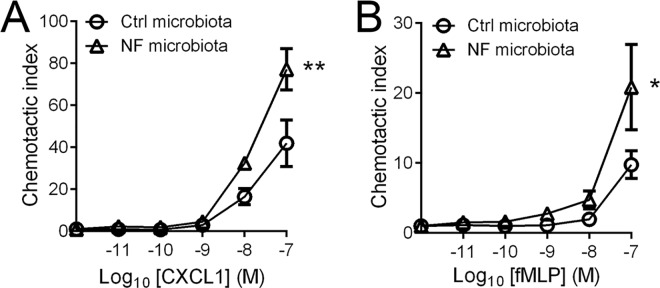
Enhanced migratory behaviour of neutrophils is maintained after microbiota transfer. Neutrophils of GF mice reconstituted with microbiota from SPF mice fed on Ctrl or NF diet were analyzed for chemotaxis towards increasing concentration of CXCL1 (**A**) and fMLP (**B**). N ≥ 4 independent *in vitro* experiments performed in duplicates, **p < 0.01, *p < 0.05 vs Ctrl diet, 2-way ANOVA.

To examine if the migratory behaviour of neutrophils relates in any way to composition and function of a microbiota shaped by diet, or the diet itself, we transferred the microbiota of SPF wildtype mice that were fed on either Ctrl or NF diet into GF mice. It is of note that the GF mice were maintained with a normal chow diet. Following two rounds of microbiota reconstitution into GF mice, we found that the neutrophils of GF mice given the microbiota shaped by the NF diet showed increased chemotaxis towards a gradient of CXCL1 (**Fig 7A**) and fMLP (**Fig 7B**). This data strongly suggests that the composition and function of a microbiota shaped by a NF diet, and not the actual content of the diet, contributed to the elevated migration of neutrophils.

## Discussion

Being largely unexplored and under-appreciated until the last couple of years, it is beginning to emerge that the gut microbiota plays an intricate and pivotal role for gut and immune homeostasis. The composition of commensal microbial communities is profoundly affected by diet and other lifestyle factors. In fact, commensal bacteria and their metabolites are critical in regulating the development, homeostasis and function of innate and adaptive immunity [1–4,8]. In this study, we tested the hypothesis that the lack of dietary fibre intake or an absence of the major receptor important in responding to the beneficial metabolites of fibre, GPR43, affected neutrophil recruitment during acute inflammation. Our study adopted animal models to mimic the increased bacterial translocation that follows elevated intestinal permeability seen in many acute and chronic inflammatory intestinal diseases. Here, we showed that neutrophil migration was closely connected to fibre in the diet. This suggests that dietary fibre, its metabolites or metabolite-sensing receptors may find use in the regulation of certain pathologic conditions such as peritonitis.

Neutrophil recruitment is instrumental for normal immune responses, however poorly regulated recruitment may contribute to unresolved or exacerbated inflammation. The high level of GPR43 expression on neutrophils [23] suggests 1) GPR43 is important for the proper functioning and migration of neutrophils in an inflammatory response; 2) The GPR43-mediated neutrophil function is likely to be dependent on the various levels of SCFAs in the microenvironment along the intestine, or in the blood; 3) The interaction of SCFA-GPR43 on neutrophils may play an immune modulation role at the intestinal level, inhibiting acute bacterial transmigration, but also an anti-inflammatory role in chronic inflammatory responses. Indeed, the immunomodulating effects of SCFAs produced following microbiota fermentation of fibre have been shown to mediate through GPR43 [12], and play an anti-inflammatory role in chronic inflammatory responses [9,15]. Mice deficient in GPR43 (*Gpr43*^*−/−*^mice) displayed exacerbated inflammation following DSS-induced colitis that is likely to be contributed at least in part by neutrophil infiltration [15]. However, another group have reported decreased DSS-induced inflammation in the colon of *Gpr43*^*−/−*^mice [24], whilst others indicated that both delayed and exaggerated immune responses can occur if SCFA signals are deficient [25]. The exaggerated immune responses that occur at later time points may be caused by delayed immune responses to the subsequent increased bacterial load in the intestinal tissue. Therefore, different interpretations of experimental outcomes are likely and it is largely dependent on the experimental models and time points chosen.

Previously, SCFAs have been shown to elicit neutrophil migration in Boyden chambers *in vitro* and in sterile air-pouch models *in vivo* [24,26]. However, this effect of SCFAs is still relatively poorly characterised and has usually involved preparations of neutrophils which have already been exposed to inflammatory mediators (e.g. derived via inflammatory peritoneal exudates) or in assays which are unable to resolve effects on adhesion, motility or direction-sensing. Here, we isolated neutrophils from the bone marrow of untreated animals and found that neutrophils from *Gpr43*^*−/−*^mice showed increased chemotaxis towards CXCL1, and unexpectedly LPS [27]. These results from *in vitro* chemotaxis assay are consistent with a previous study which described GPR43-deficient neutrophils being “hyper-migratory” towards bacterial product (fMLP) and to the complement fragment C5a [15]. Furthermore, the fact that this elevated migration of neutrophils was conserved following two rounds of microbiota transfer strongly indicates the importance of microbiota composition in the regulation of neutrophil trafficking.

One of the factors involved in the recruitment of neutrophils is L-selectin. A recent study has shown that the cytoplasmic tail of L-selectin is required for downstream signalling and slow leukocyte rolling [28]. Shedding of L-selectin was observed shortly after adhesion to endothelial cells and may be essential for neutrophil migration [29]. The precise role of SCFAs in the regulation of L-selectin and its contribution to rolling velocity is currently debatable. Two studies from independent groups published in the same year reported contrasting results. On one hand, it was reported that SCFAs could potentially influence the shedding of neutrophil L-selectin via GPR43 [24]. In contrast, SCFA treatment was shown to increase L-selectin expression on the neutrophil surface and L-selectin mRNA levels [26]. Nevertheless, our experiments suggest that the basal expression and PMA-induced shedding of L-selectin are comparable between wildtype and *Gpr43*^*−/−*^neutrophils *in vitro*, but SCFAs and GPR43 are involved in at least two steps of neutrophil recruitment *in vivo* following LPS challenge.

With the use of intravital microscopy and an endotoxin model of acute inflammation, we observed exacerbated neutrophil rolling and arrest within the intestinal vasculature of *Gpr43*^*−/−*^mice as early as 1 h following LPS, suggesting that the SCFAs-GPR43 axis is critical in restricting overt neutrophil response following such inflammatory stimuli. Specifically, our data suggests GPR43 is involved in modulation of the initial tethering and rolling of neutrophils at early time points following inflammatory stimuli *in vivo*. Following prolonged (4 h) LPS exposure, neutrophils from GPR43-deficient or fibre-deficient wildtype mice developed prolonged rolling interactions with the vessel wall. In fact, we showed in both our *in vitro* and *in vivo* experiments that GPR43-deficient neutrophils are highly responsive to LPS. We also demonstrated that GPR43-deficient neutrophils have transmigrated into the lamina propria of the small intestine. This finding was further confirmed in the peritonitis model experiments, which clearly demonstrated GPR43 as a metabolite-sensing receptor responsible for modulating the neutrophil migratory effects of acetate after an acute fMLP-induced inflammatory response. Together, these results suggest that acetate has potential therapeutic utility in conditions where neutrophil influx needs to be controlled.

There is currently no single ideal experimental model that completely recapitulates human IBD, because patients with IBD present a heterogeneous spectrum of pathological features, involving the participation of a diverse range of innate and adaptive immune effectors [30,31]. Despite this, a common shared mechanism is disruption of the epithelial barrier, and increased exposure of the host immune system to intestinal microflora. In fact, analysis of granulomas revealed the presence of *E*. *coli* DNA in ~80% of Crohn's disease patients, suggesting that mucosal-infiltrated bacteria may have a role in the inflammatory process [32]. Indeed, a pathological hallmark of active IBD is a strong migration of neutrophils into the mucosa, which are characteristically found in the lamina propria and in the epithelial layer of IBD patients [33]. It was proposed that this massive infiltration of neutrophils in the gut functions to kill luminal microbes that have translocated across the epithelium and invaded the mucosa, but the relative contributions of neutrophils to the pathogenesis of IBD is controversial with some studies describing a beneficial role, yet, others reporting a pathological role [34,35]. These contrary results provoke the notion that neutrophils may play a dual and complex immune modulating role in intestinal inflammation. Clearly, there needs to be better methodology to identify and further investigate the dynamic process of neutrophil transmigration during intestinal inflammation *in vivo*.

Recent studies have shown dietary fibre positively shapes the composition of the gut microbiota and immunity, yet the underlying mechanisms through which dietary fibre modulates and protects the host from acute intestinal inflammation remain to be elucidated. Our study utilised animal models to emulate the elevated bacterial translocation that follows increased intestinal permeability seen in many acute and chronic inflammatory intestinal diseases. We have used novel imaging techniques to demonstrate that SCFAs produced through bacterial fermentation of dietary fibre contribute directly to the regulation of host intestinal neutrophil responses, through the function of anti-inflammatory chemoattractant receptor GPR43. Consistent with previous studies, we report here that mice deficient in GPR43 respond differently in models of intestinal inflammation, suggesting that this system may provide a foundation for the development of novel and targeted therapies for treating acute and chronic intestinal inflammatory diseases.

## Supporting Information

S1 Movie Intravascular neutrophil behaviour in wildtype mice following LPS challenge. Intravital spinning-disk confocal microscopy was utilised to examine the behaviour of intravascular neutrophils in the small intestine of wildtype mice at 4 h after LPS challenge. Neutrophils were visualised using PE-conjugated anti-Ly6G (red) and endothelium was visualised with FITC-conjugated anti-CD31 (green). The time lapse was recorded at 25 fpm and exported to video at 7 fps.
(MOV)

S2 Movie Intravascular neutrophil behaviour in *Gpr43*^*−/−*^mice following LPS challenge. Intravital spinning-disk confocal microscopy was utilised to examine the behaviour of intravascular neutrophils in the small intestine of *Gpr43*^*−/−*^mice at 4 h after LPS challenge. Neutrophils were visualised using PE-conjugated anti-Ly6G (red) and endothelium was visualised with FITC-conjugated anti-CD31 (green). The time lapse was recorded at 25 fpm and exported to video at 7 fps.
(MOV)
